# Protocol to reconstruct an *in vitro* 3D full-thickness skin equivalent model with collagen I in 6- and 12-well inserts

**DOI:** 10.1016/j.xpro.2025.103658

**Published:** 2025-03-04

**Authors:** Khek-Chian Tham, Seong Soo Lim, Carine Bonnard, John E.A. Common

**Affiliations:** 1 A∗STAR Skin Research Labs (A∗SRL), Agency for Science, Technology and Research (A∗STAR), 11 Mandalay Road, #17-01 Clinical Sciences Building, Singapore 308232, Singapore; 2 Asian Skin Biobank, Skin Research Institute of Singapore (SRIS), 11 Mandalay Road, #17-01 Clinical Sciences Building, Singapore 308232, Singapore; 3 Translational and Clinical Research Institute and NIHR Newcastle Biomedical Research Centre, Newcastle University, Newcastle upon Tyne, UK

**Keywords:** Cell Biology, Cell culture, Cell Differentiation, Tissue Engineering

## Abstract

*In vitro* 3D full-thickness reconstituted human skin has high physiological relevance due to the presence of differentiation features often lacking in 2D cell cultures. Here, we present a protocol to reconstruct a 3D skin model using human fibroblasts, keratinocytes, and rat-tail collagen I. We describe steps for cell expansion, the casting of cellular and acellular layers, seeding keratinocytes, the air lifting of culture, and incubation. We also demonstrate the use of chitosan to prevent tissue contraction in 12-well inserts.

For complete details on the use and execution of this protocol, please refer to Robinson et al.^1^^,^^2^

## Highlights

• Protocol for 3D skin reconstruction using fibroblasts and keratinocytes
• Instruction for 2D expansion of human fibroblast and human keratinocyte cells
• Differential steps for 6-well and 12-well format of 3D skin culture

## Abstract (editor highlights)

Publisher’s note: Undertaking any experimental protocol requires adherence to local institutional guidelines for laboratory safety and ethics.

## Graphical abstract

**Figure:**
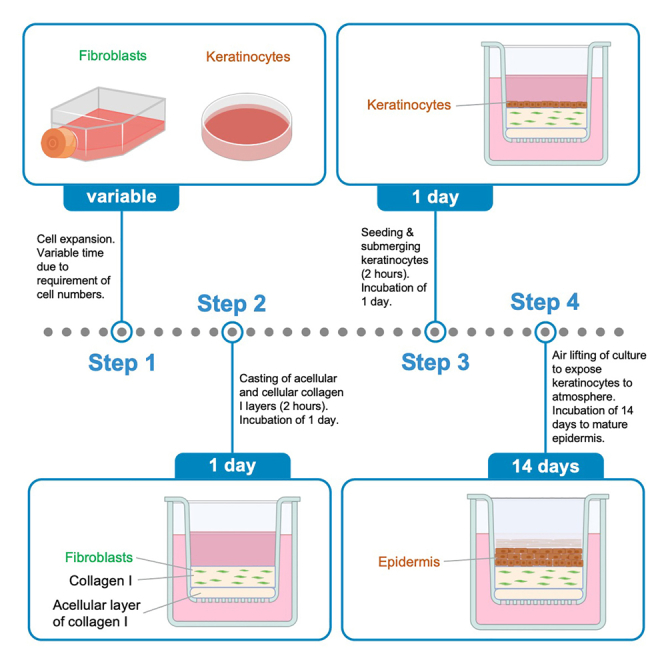

## Before you begin

**Timing: 4 weeks**

This protocol outlines the step-by-step process for generating a full-thickness 3D skin model using human fibroblasts and keratinocytes. The model is reconstructed in 6- and 12-well culture inserts, starting with the isolation of primary skin cells. Fibroblasts and keratinocytes are first expanded in 2D cultures and cryopreserved in bulk to ensure consistency across experiments. The cells can then be thawed for further 2D culture to initiate the protocol below prior to establishing the 3D skin. This approach enables the development of robust and reproducible 3D skin models for various applications, including research on skin biology and disease.

1. Prepare stock reagents of human keratinocyte medium (see materials and equipment).
2. Prepare media for mouse 3T3-J2 fibroblast, human fibroblast, and keratinocyte 2D cultures (see materials and equipment).
3. Prepare irradiated 3T3-J2 feeder cells for 2D human keratinocyte cultures.

***Note:*** 2D cultures of 3T3-J2, human fibroblasts and keratinocytes require different media.

### Institutional permissions

Human primary cells used for experiments are governed by local institutional ethical review and therefore must be approved by relevant regulators. All cells and reagents used in this protocol have been approved for use by our local ethical review panels.

### Prepare irradiated 3T3-J2 feeder cells for 2D human keratinocyte cultures

**Timing: 2–3 weeks**

**Figure 1:**
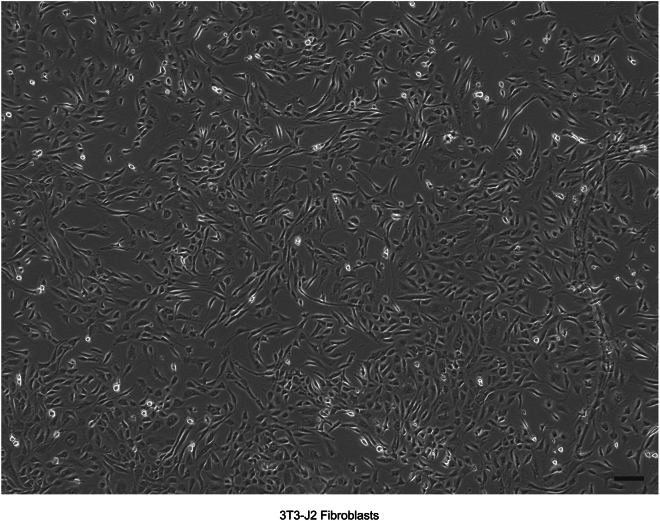
Bright-field image of 3T3-J2 fibroblasts At this near confluent density, the cells are ready for detachment and gamma irradiation. Scale bar, 150 μm.
5. Detach the cells with 10-mL 0.05% Trypsin-EDTA for each flask in a humidified incubator with 10% CO_2_ at 37°C for 5–10 min.
6. Neutralize trypsin with 10-mL 3T3-J2 culture medium pre-warmed at 37°C.
7. Spin down the cells at 1000 rpm for 5 min.
8. Discard the supernatant and resuspend the pellet with 1-mL pre-warmed 3T3-J2 culture medium.
9. Pool all cells in a 50-mL tube and top up with 30-mL pre-warmed 3T3-J2 culture medium.
10. Keep the cells in ice until irradiation.
11. Irradiate the cells with 60 Gy of gamma radiation source (Gammacell 40 Exactor).

***Note:*** This step can be replaced by treating the cells with 10 μg/mL of mitomycin C for 4 h at 37°C if gamma radiation source is not available.

4. Expand 3T3-J2 fibroblast cultures with twenty T175 flasks to near confluent density as shown (Figure 1), in a humidified incubator with 10% CO_2_ at 37°C.

12. Filter the cells through a 40-μm strainer.
13. Count the cells and freeze down 1–2 x 10^6^ cells per vial in 900-μL bovine calf serum and 100-μL DMSO.

## Key resources table

**Table:** 

REAGENT or RESOURCE	SOURCE	IDENTIFIER
**Antibodies**		

K14 (1:500)	Abcam	Cat # ab181595
Vimentin (1:100)	Leica	Cat # NCL-L-VIM-V9
K10 (1:500)	Abcam	Cat # ab76318
Ki67 (1:100)	Invitrogen	Cat # 14-5698-82
Filaggrin (1:100)	Santa Cruz	Cat # sc-66192
Loricrin (1:100)	BioLegend	Cat # 905104
Claudin-1 (1:100)	Abcam	Cat # ab140349
Secondary antibodies (1:500)	Invitrogen	Cat # A32723, A32727, A32731, A32732, A11077

**Chemicals, peptides, and recombinant proteins**		

0.5% trypsin-EDTA	Gibco	Cat # 15400054
DMEM with sodium pyruvate	HyClone	Cat # SH30243.01
DMEM without sodium pyruvate	HyClone	Cat # SH30022.01
F12	Gibco	Cat # 31765035
FBS	HyClone	Cat # SV30160.03
BCS	HyClone	Cat # SH30072.03
100 X penicillin/streptomycin	Gibco	Cat # 15140-122
Adenine	Sigma	Cat # A2786
Hydrocortisone	Sigma	Cat #H0888
Cholera toxin	Enzo	Cat # BML-G117-001
Insulin	Sigma	Cat #I2643
Transferrin	Sigma	Cat #T2036
Triiodothyronine	Sigma	Cat #T6397
EGF	Sigma	Cat #E9644
Y-27632	Tocris	Cat # 1254
Chitosan	Sigma	Cat # 448877
Rat-tail collagen I	Corning	Cat # 354249
10 X DMEM	Sigma	Cat #D2429
Sodium bicarbonate	Sigma	Cat #S5761
1 N sodium hydroxide	1^st^ Base	Cat # BUF-1115
1 M HEPES	HyClone	Cat # SH30237.01
16% formaldehyde	Thermo Scientific	Cat # 28908
1 X PBS	Gibco	Cat # 20012027
DAPI	Invitrogen	Cat #R37606

**Experimental models: Cell lines**		

3T3-J2	Kerafast	Cat # EF3003
Primary human skin fibroblasts	Asian Skin Biobank, Skin Research Institute of Singapore (SRIS)	Donor ID # 12-S-Fibro-012, 14-S-Fibro-001, 17-S-Fibro-017, 17-S-Fibro-028, 13-S-Fibro-017
Primary human skin keratinocytes	Asian Skin Biobank, Skin Research Institute of Singapore (SRIS)	Donor ID # 12-S-Kera-012, 14-S- Kera-001, 17-S- Kera-017, 17-S- Kera-028, 13-S-Kera-017

**Other**		

0.2-μm syringe filter	Sartorius	Cat # 16534-K
T75 flask	Nunc	Cat # 156499
T175 flask	Nunc	Cat # 159910
10-cm dish	Nunc	Cat # 150466
6-well inserts	Corning	Cat # 353102
6-well deep well plates	Corning	Cat # 355467
12-well inserts	Greiner	Cat # 665610
12-well deep-well plates	Greiner	Cat # 665110
Low liquid retention 1-mL pipette tips	Vertex	Cat # 4347NSF
40-μm strainer	Corning	Cat # 352340
Scalpel blade	B. Braun	Cat # BB511
Single edge stainless steel blade	Electron Microscopy Sciences	Cat # 71970

## Materials and equipment

**Table:** 3T3-J2 fibroblast medium

Reagent	Final concentration	Amount
DMEM without sodium pyruvate	N/A	500 mL
BCS	9%	50 mL
100 X penicillin/streptomycin	0.9 X	5 mL
**Total**	N/A	**555 mL**

Store at 4°C for up to 1 month.

**Table:** Human fibroblast medium

Reagent	Final concentration	Amount
DMEM with sodium pyruvate	N/A	500 mL
FBS	9%	50 mL
100 X penicillin/streptomycin	0.9 X	5 mL
**Total**	N/A	**555 mL**

Store at 4°C for up to 1 month.

**Table:** Human keratinocyte medium

Reagent	Final concentration	Amount
DMEM with sodium pyruvate	N/A	333 mL
F12	N/A	111 mL
FBS	10%	50 mL
100 X penicillin/streptomycin	1 X	5 mL
9.72 mg/mL adenine	0.0243 mg/mL	1.25 mL
200 μg/mL hydrocortisone	0.4 μg/mL	1 mL
1 x 10^-7^ M cholera toxin	1 x 10^-10^ M	0.5 mL
5 mg/mL insulin	5 μg/mL	0.5 mL
5 mg/mL transferrin	5 μg/mL	0.5 mL
2 x 10^-6^ M triiodothyronine	2 x 10^-9^ M	0.5 mL
10 μg/mL EGF	10 ng/mL	0.5 mL
10 mM Y-27632	10 μM	0.5 mL
**Total**	N/A	**504.25 mL**

Sterilize stock reagents of adenine, hydrocortisone, cholera toxin, insulin, transferrin, triiodothyronine, EGF and Y-27632 with 0.2-μm syringe filters and store at −20°C for up to 1 year.

Store final prepared medium at 4°C for up to 1 month.

**Table:** 10X reconstitution buffer

Reagent	Final concentration	Amount
Sodium bicarbonate	26.19 mM	0.11 *g*
1 N sodium hydroxide	0.005 N	0.25 mL
1 M HEPES	0.02 M	1 mL
Sterile water	N/A	48.75 mL
**Total**	N/A	**50 mL**

Sterilize buffer with a 0.2-μm syringe filter and store at 4°C for up to 6 months.

## Step-by-step method details

**Figure 2:**
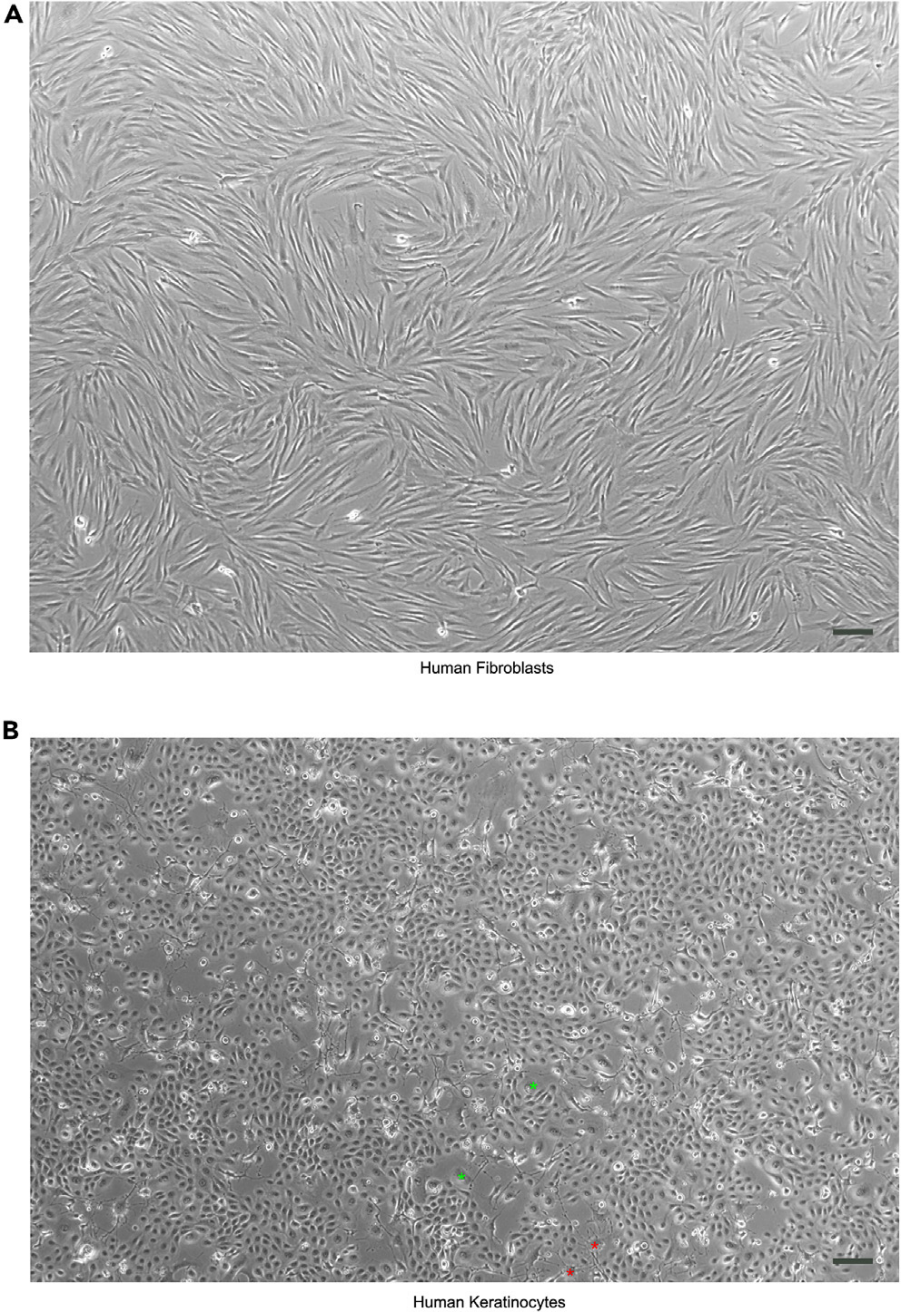
Bright-field image of human fibroblasts and keratinocytes Both images show optimal density for passaging. Typically, (A) human fibroblasts appear as long and slender, while (B) proliferative human keratinocytes appear as small, round and adhering with each other. The irradiated 3T3-J2 feeder cells are labeled with red asterisks, while the differentiated keratinocytes are labeled with green asterisks. Scale bar, 150 μm.

To reconstruct the 3D skin equivalent model, grow sufficient number of human fibroblasts for the preparation of dermal equivalent layer with collagen I and chitosan (chitosan is required for 12-well format only to reduce collagen contraction), grow sufficient human keratinocytes to seed onto the dermal equivalent layer for epidermal differentiation and stratification induced by air lifting. Human fibroblasts and human keratinocytes should be grown to approximately 80% confluency to maximize cell numbers, and maintain cell health and proliferative potential (Figure 2). Culturing human fibroblasts and human keratinocytes, as described below, will take approximately 3–5 days to reach desired confluency (Figure 2). One T75 of human fibroblast culture will yield 1–2 million cells, and one 10 cm dish of human keratinocytes will yield 5–6 million. One 6-well insert requires 0.75 x 10^6^ human fibroblasts and 1 x 10^6^ human keratinocytes, while one 12-well insert needs 0.1875 x 10^6^ human fibroblasts and 0.25 x 10^6^ human keratinocytes.

### Expand human fibroblasts for the preparation of 3D dermal equivalent layer

**Timing: variable (depending on experimental scale)**

The expanded human fibroblasts will be used in the reconstruction of 3D dermal equivalent layer with collagen I.

1. Thaw cryovial in 37°C water bath and mix gently with 10-mL pre-warmed human fibroblast culture medium.
2. Spin down the cells at 1000 rpm for 5 min.
3. Discard the supernatant and resuspend the pellet with 1-mL pre-warmed human fibroblast culture medium.
4. Seed 0.5–1 x 10^6^ cells into one T75 flask filled with 12-mL pre-warmed human fibroblast culture medium.
5. Change culture medium every 2 days.
6. Do not grow the cells at confluent density (Figure 2A) for more than a day.
7. Detach the cells with 5-mL 0.05% Trypsin-EDTA in a humidified incubator with 5% CO_2_ at 37°C for 5–10 min.
8. Neutralize trypsin with 5-mL pre-warmed human fibroblast culture medium.
9. Spin down the cells at 1000 rpm for 5 min.
10. Discard the supernatant and resuspend the pellet with pre-warmed human fibroblast culture medium.
11. Expand the cells to desired amount.

### Expand human keratinocytes for the preparation of 3D epidermal equivalent layer

**Timing: variable (depending on experimental scale)**

Culturing of human keratinocytes may begin 1–3 days later than culturing of human fibroblasts due to faster growth rate of keratinocytes. The expanded human keratinocytes will be used in the reconstruction of 3D epidermal equivalent layer. Human keratinocytes are expanded in the presence of Y-27632 to allow for long term proliferative potential (Chapman et al.^3^). We typically use human keratinocytes between passage 3 and 10 to ensure consistency in protocol success.

12. Thaw irradiated 3T3-J2 in 37°C water bath and mix gently with 10-mL pre-warmed 3T3-J2 medium.
13. Spin down the cells at 1000 rpm for 5 min.
14. Discard the supernatant and resuspend the pellet with 1-mL pre-warmed 3T3-J2 medium.
15. Seed 0.4 x 10^6^ irradiated 3T3-J2 fibroblasts into one 10-cm dish filled with 10-mL pre-warmed 3T3-J2 culture medium.
16. Next day, thaw human keratinocytes in 37°C water bath and mix gently with 10-mL pre-warmed human keratinocyte culture medium.
17. Spin down the cells at 1000 rpm for 5 min.
18. Discard the supernatant and resuspend the pellet with 1-mL pre-warmed human keratinocyte culture medium.
19. Seed 0.5–1 x 10^6^ cells into one 10-cm dish pre-seeded with 0.4 x 10^6^ irradiated 3T3-J2 fibroblasts and filled with 10-mL pre-warmed human keratinocyte culture medium.
20. Change culture medium every 2 days.
21. Do not grow the cells at confluent density (Figure 2B) for more than a day.
22. Detach the cells with 5-mL 0.1% Trypsin-EDTA in a humidified incubator with 5% CO_2_ at 37°C for 5–10 min.
23. Filter the cells through a 40-μm strainer to remove irradiated feeder cells and differentiated keratinocytes.

***Note:*** This step is only necessary before seeding the cells onto the dermal equivalent layer, not necessary for routine passaging.

24. Neutralize trypsin with 10-mL pre-warmed human keratinocyte culture medium.
25. Spin down the cells at 1000 rpm for 5 min.
26. Discard the supernatant and resuspend the pellet with 1-mL pre-warmed human keratinocyte culture medium.
27. Expand the cells to desired amount.

***Note:*** Typically, one 10-cm dish at confluent density produces at least 5 x 10^6^ cells.

### Preparation of 3D dermal equivalent layer with collagen I and human fibroblasts

**Timing: 1 day**

**Figure 3:**
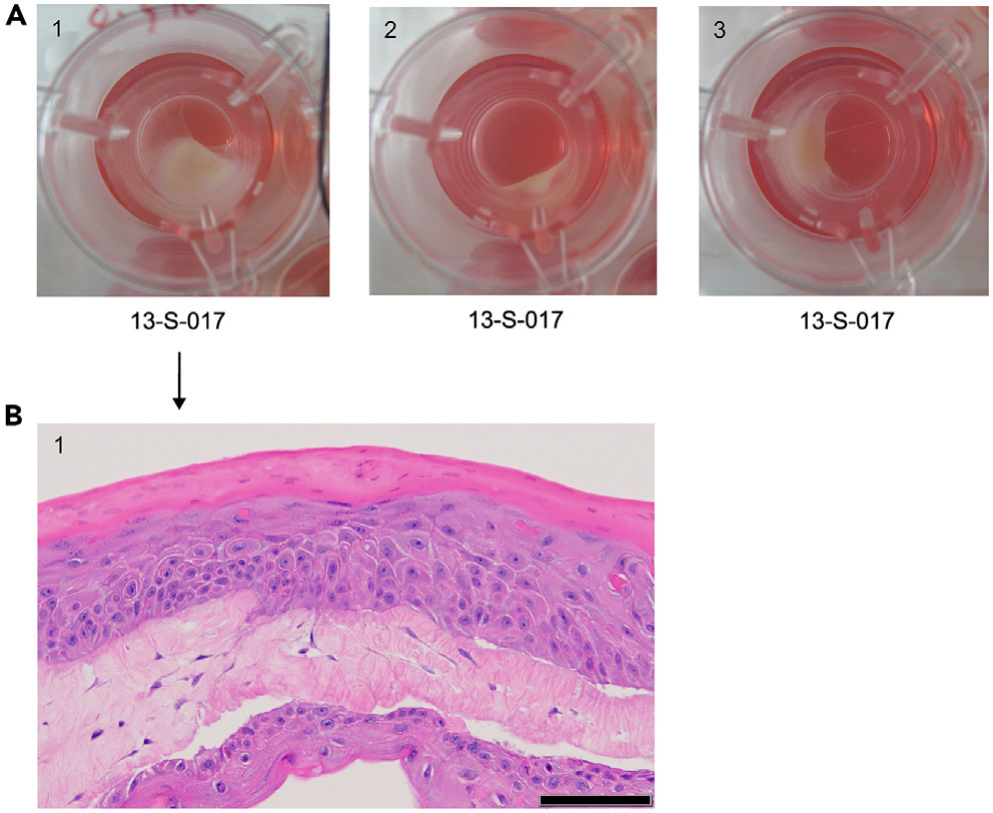
Severe contraction accompanied with abnormal skin morphology occurs with 12-well insert (A) Top view images of three skin tissues reconstructed with 12-well inserts cultured in the absence of chitosan on the 14^th^ day of air lifting. 13-S-017 is the de-identified donor ID. (B) Cross-section H&E staining of the least contracted tissues. Scale bar, 100 μm.

*In vitro* 3D full-thickness skin can be reconstructed in 6- or 12-well inserts and deep well plates. Due to the nature of organotypic tissue contraction, casting a layer of chitosan-collagen I matrix directly on the insert membrane below the dermal equivalent layer prevents contraction in 12-well format. In the absence of chitosan, severe contraction can occur in 12-well inserts (Figure 3A) accompanied with abnormal and variable skin morphology (Figure 3B).

**Figure:**
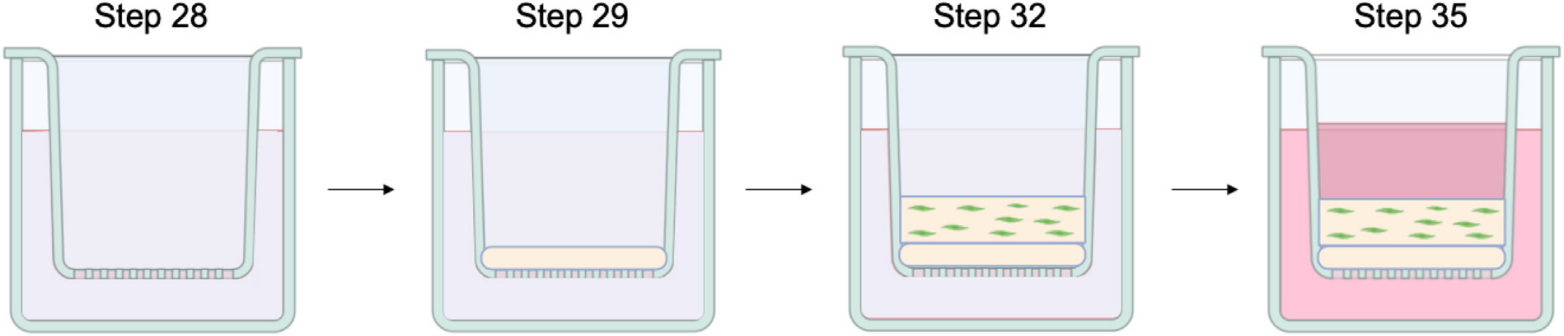

28. Place inserts into deep well plates using a sterile forceps.
29. Prepare an acellular matrix layer to reduce tissue contraction.
  a. Add 1-mL of collagen I mixture per 6-well insert.
    **Table:** Collagen I mixture

Reagent	Final concentration	Amount
Sterile water	N/A	Top up to 1 mL
10 X reconstitution buffer	1 X	0.1 mL
10 X DMEM	1 X	0.1 mL
Rat-tail collagen I	4 mg/mL	variable
1N sodium hydroxide	N/A	variable
**Total**	N/A	**1 mL**

    **CRITICAL:** Keep all reagents and mixture in ice. Collagen I and mixture solidify at room temperature.
    ***Note:*** Add the reagents according to the order of the table.
    ***Note:*** Rat-tail collagen I stock concentration varies according to batch. Use low liquid retention 1-mL pipette tips for accurate pipetting of viscous collagen. Swirl the reagents properly with additional of every mL of collagen I to ensure thorough mixing.
    ***Note:*** Add sodium hydroxide gradually to achieve mixture pH with faint red appearance. Different batches of collagen I requires different amount of sodium hydroxide. As an example, 1-mL mixture requires ∼14 μL of 1N sodium hydroxide.
    ***Note:*** After adding all reagents, keep the mixture in ice for a few minutes, such that tiny bubbles float to the surface of the mixture that impact the quality of the final skin. Avoid surface bubbles during pipetting.
    ***Note:*** Upscale the recipe according to the total number of samples.
  b. Add 250-μL of chitosan-collagen I mixture in each 12-well insert.
    **Table:** Chitosan-collagen I mixture

Reagent	Final concentration	Amount
Sterile water	N/A	Top up to 1 mL
10 X reconstitution buffer	1 X	0.1 mL
10 X DMEM	1 X	0.1 mL
Rat-tail collagen I	4 mg/mL	variable
20 mg/mL chitosan	2.5 mg/mL	0.125 mL
1N sodium hydroxide	N/A	variable
**Total**	N/A	**1 mL**

    ***Note:*** The table notes of collagen I mixture apply to the preparation of chitosan-collagen I mixture.
    ***Note:*** It is not necessary to use chitosan for 6-well format due to bigger tissue size.
30. Gently tilt and rotate the mixture in 6-well insert to mitigate tissue contraction.

***Note:*** It is not necessary to tilt and rotate the mixture in 12-well format because the surface area is too small.

31. Incubate the mixture in a humidified incubator with 5% CO_2_ at 37°C for 30 min, maximum 60 min.
32. Prepare collagen I-fibroblast layer on top of the acellular layer.
  a. Add 2-mL collagen I-fibroblast mixture in each 6-well insert.
  b. Add 500-μL collagen I-fibroblast mixture in each 12-well insert.

**Table:** Collagen I-fibroblast mixture

Reagent	Final concentration	Amount
Sterile water	N/A	Top up to 2 mL
10 X reconstitution buffer	1 X	0.2 mL
10 X DMEM	1 X	0.2 mL
Human fibroblasts	0.75 x 10^6^	pellet
Rat-tail collagen I	4 mg/mL	variable
1N sodium hydroxide	N/A	variable
**Total**	N/A	**2 mL**

***Note:*** The table notes of collagen I mixture apply to collagen I-fibroblast mixture.

***Note:*** Before adding viscous collagen I, prepare a mix of water, 10 X buffer and 10 X DMEM to resuspend the human fibroblast pellet.

**CRITICAL:** Do not resuspend the pellet with water only.

33. Tilt and rotate the mixture in 6-well insert to mitigate tissue contraction.

***Note:*** It is not necessary to tilt and rotate the mixture in 12-well format because the surface area is too small.

34. Incubate the mixture in a humidified incubator with 5% CO_2_ at 37°C for 30 min, maximum 60 min.
35. Add pre-warmed human fibroblast culture medium into the inserts and the deep wells.
  a. Add 6-mL culture medium into the 6-well insert and 10-mL culture medium into the well.
  b. Add 500-μL culture medium into the 12-well insert and 5-mL culture medium into the well.

***Note:*** Avoid generating bubbles below the insert membrane.

36. Incubate the tissues in a humidified incubator with 5% CO_2_ at 37°C overnight.

### Preparation of 3D epidermal equivalent layer with human keratinocytes

**Timing: 1 day**

Keratinocytes are the major cell type that form a protective barrier against external assaults. Keratinocytes have donor to donor variability regarding cell proliferation rates. This should be tracked during the 2D expansion step. Following the steps below using the correct number of keratinocytes harvested for 2D cultures will result in a complete stratified epidermis irrespective of the donor-to-donor variability in proliferation.

37. Aspirate human fibroblast culture medium from the inserts and deep wells.

**CRITICAL:** Avoid touching the 3D dermal equivalent layer during the suction.

38. Add human keratinocyte culture medium onto the 3D dermal equivalent layer.
  a. Add 1-mL culture medium into the 6-well insert.
  b. Add 100-μL culture medium into the 12-well insert.
39. Seed human keratinocytes in human keratinocyte culture medium onto the 3D dermal equivalent layer.
  a. Seed 1 x 10^6^ cells in 1-mL culture medium into a 6-well dermal equivalent layer.
  b. Seed 0.25 x 10^6^ cells in 100-μL culture medium into a 12-well dermal equivalent layer.
40. Shake the plates gently and horizontally for even cell spreading.
41. Incubate the cultures in a humidified incubator with 5% CO_2_ at 37°C for 30 min.

***Note:*** Shake the plates side-to-side every 5–10 min during the incubation to enhance even cell spreading. Orbital shaking can result in cells concentrating in the center of the insert impacting of final skin quality.

42. Add additional human keratinocyte media into the inserts and the deep wells.
  a. Add gently 4-mL culture medium into the 6-well insert and 10-mL culture medium into the well.
  b. Add gently 300-μL culture medium into the 12-well insert and 5-mL culture medium into the well.

***Note:*** Avoid generating bubbles below the insert membrane.

43. Incubate the cultures in a humidified incubator with 5% CO_2_ at 37°C overnight.

### Induction of 3D epidermal differentiation and stratification

**Timing: 14 days**

Exposing the keratinocytes adhered to the 3D dermal equivalent layer to air promotes terminal differentiation program. Ultimately, the 3D epidermal layer stratifies into strata basale, spinosum, granulosum, and the outermost stratum corneum.

44. Prepare human keratinocyte culture medium without EGF and Y-27632.

***Note:*** EGF and Y-27632 are not required for the air lifting stage.

45. Aspirate human keratinocytes culture medium from the inserts and deep wells.

**CRITICAL:** Avoid touching the 3D dermal equivalent layer during the suction.

46. Add the culture medium prepared in step 44 into the deep wells only.
  a. Add 9-mL culture medium into the 6-well deep well.
  b. Add 4-mL culture medium into the 12-well deep well.

***Note:*** Avoid generating bubbles below the insert membrane.

47. Refresh the culture medium in the deep wells every 2 days.

### 3D skin tissue processing for histology

**Figure:**
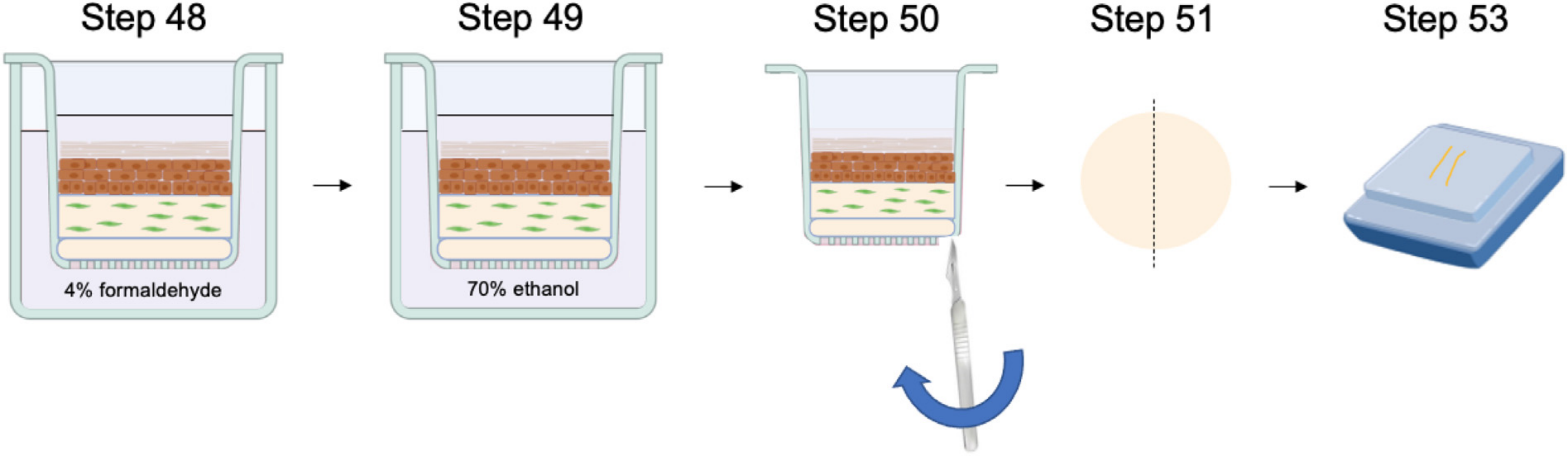

**Timing: 2 days**

48. Fix the skin tissue in inserts with 4% formaldehyde overnight at room temperature on the 14^th^ day of air lifting.

***Note:*** Dilute 16% formaldehyde stock with 1 X PBS.

***Note:*** Corneum layer is apparent on the 10^th^ day of air lifting.

49. Change to 70% ethanol and incubate overnight at room temperature.
50. Cut the membrane adhered with skin tissues along the insert contour with a scalpel blade.

***Note:*** Avoid tearing the membrane and make clean cut.

51. Cut the membrane adhered with skin tissues into two halves at the center with a single edge blade.

***Note:*** Avoid tearing the membrane and make clean cut.

52. Remove the membrane carefully from the skin tissues with 2 forceps.
53. Transfer the tissues in 70% ethanol for downstream processing into paraffin embedded blocks for sectioning followed by hematoxylin and eosin (H&E) and immunohistochemical staining.

## Expected outcomes

**Figure 4:**
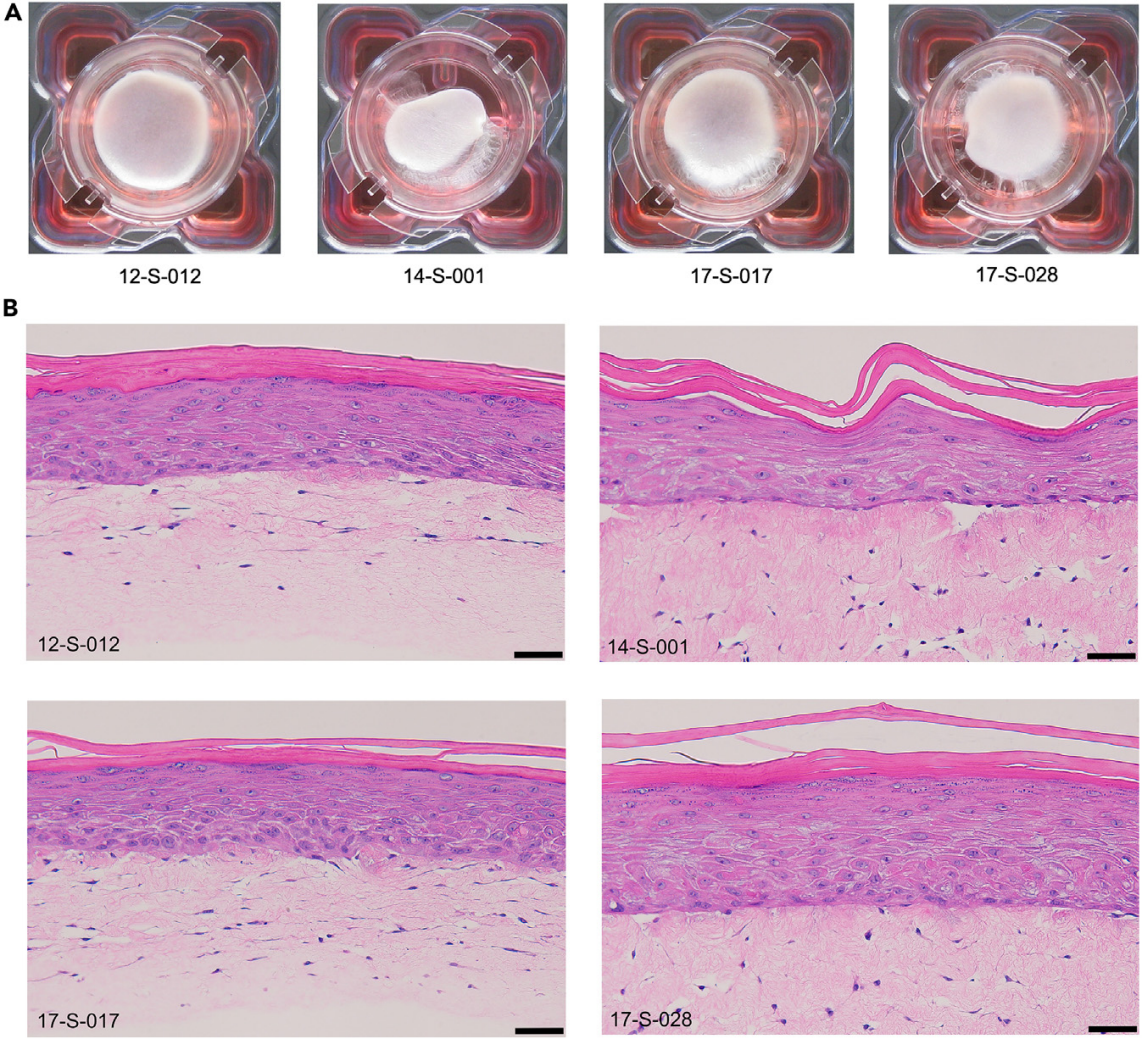
3D skin models reconstructed with donor-matched human fibroblasts and keratinocytes in 6-well inserts (A) Top view images of the 6-well tissues cultured with cells derived from 4 different human donors on the 14^th^ day of air lifting. 12-S-012, 14-S-001, 17-S-017 and 17-S-028 are the de-identified donor IDs. (B) H&E staining of the 6-well cross section tissues. Scale bar, 50 μm.

**Figure 5:**
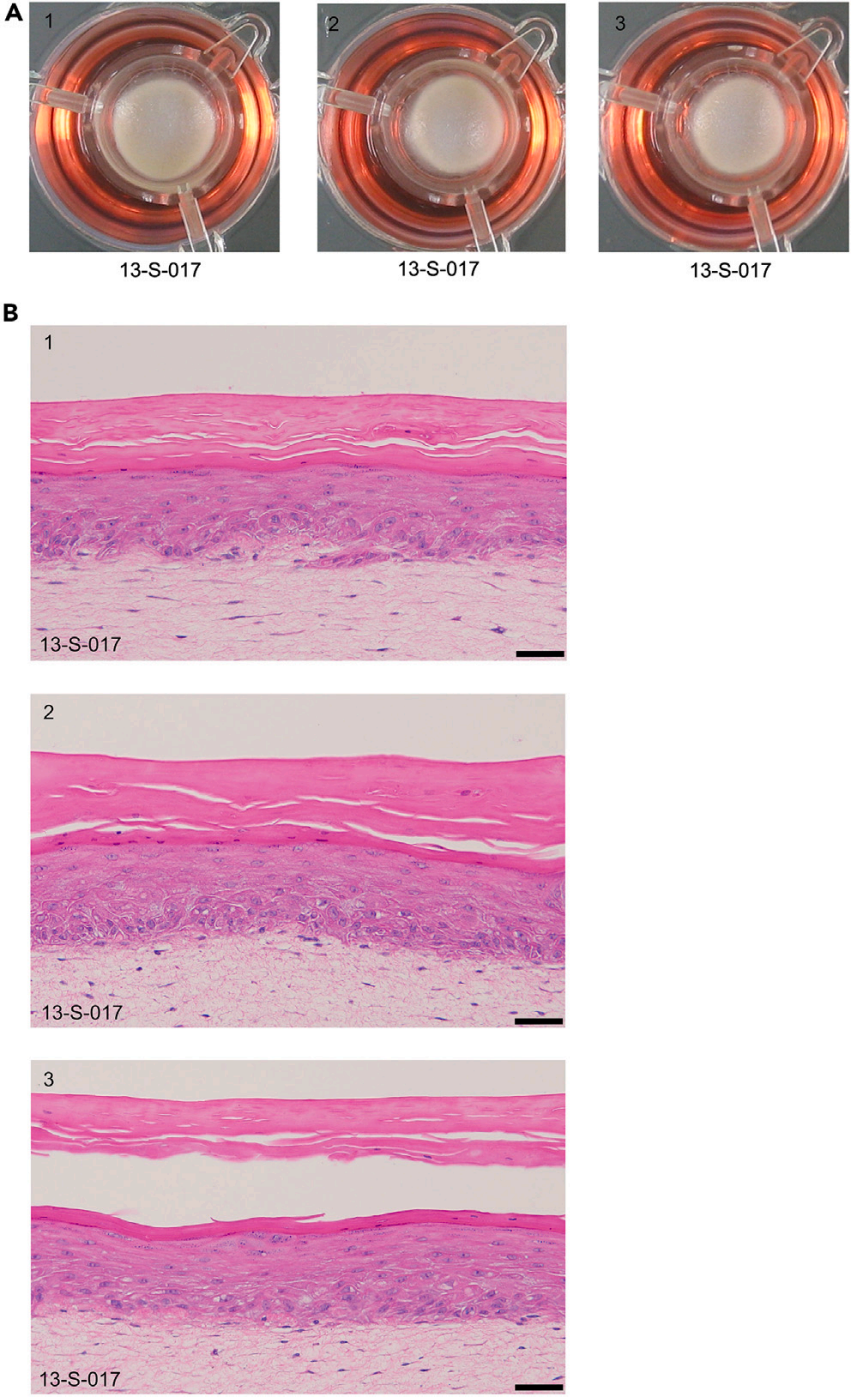
3D skin models reconstructed in 12-well inserts with chitosan-collagen I layer (A) Top view images of three skin tissues reconstructed with 12-well inserts in the presence of chitosan on the 14^th^ day of air lifting. 13-S-017 is the de-identified donor ID. (B) Cross-section H&E staining of the 3 replicate tissues. Scale bar, 50 μm.

We observe that donor-matched human fibroblasts and keratinocytes derived from different human donors display varying degree of tissue contraction in 6-well 3D skin cultures (Figure 4A). However, the contraction does not affect epidermal differentiation as shown by H&E staining (Figure 4B). Similarly, the reconstructed skin tissue using 12-well inserts (Figure 5A) largely prevent tissue contraction with chitosan and display normal skin morphology as shown by H&E staining (Figure 5B).

**Figure 6:**
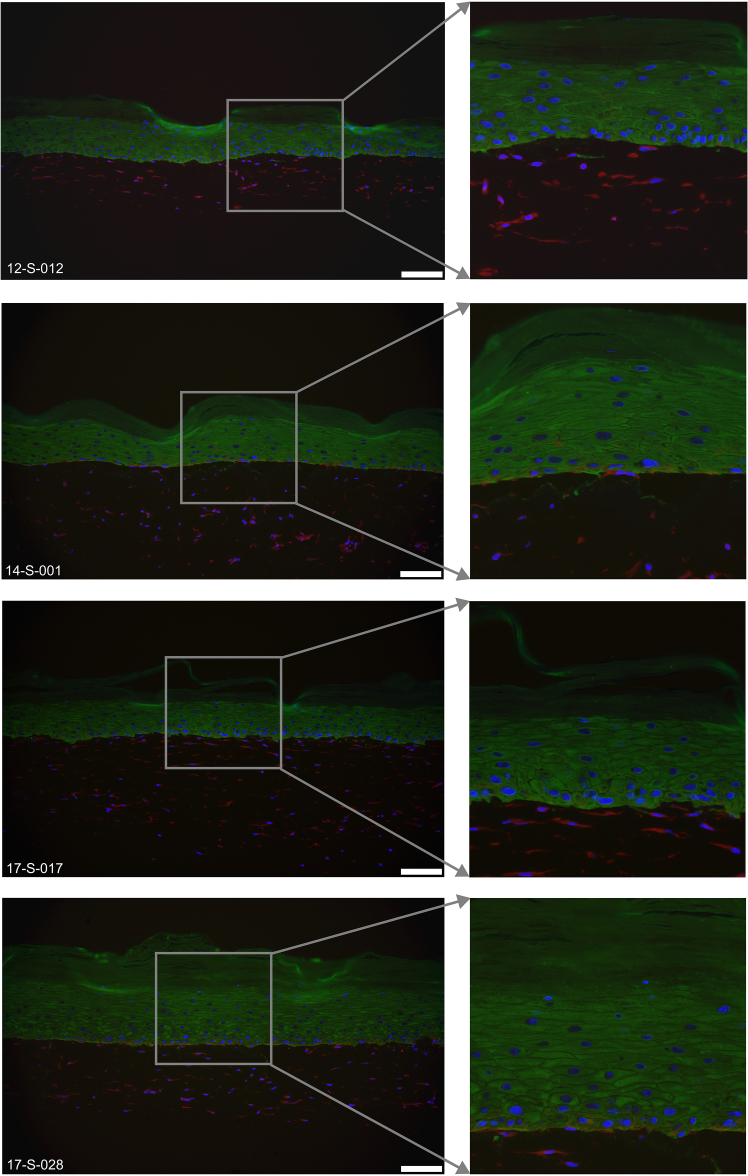
Representative immunofluorescence images of K14 (green) and Vimentin (red) expressed in the 6-well tissues 12-S-012, 14-S-001, 17-S-017 and 17-S-028 are the deidentified donor IDs. Scale bar, 100 μm.

**Figure 7:**
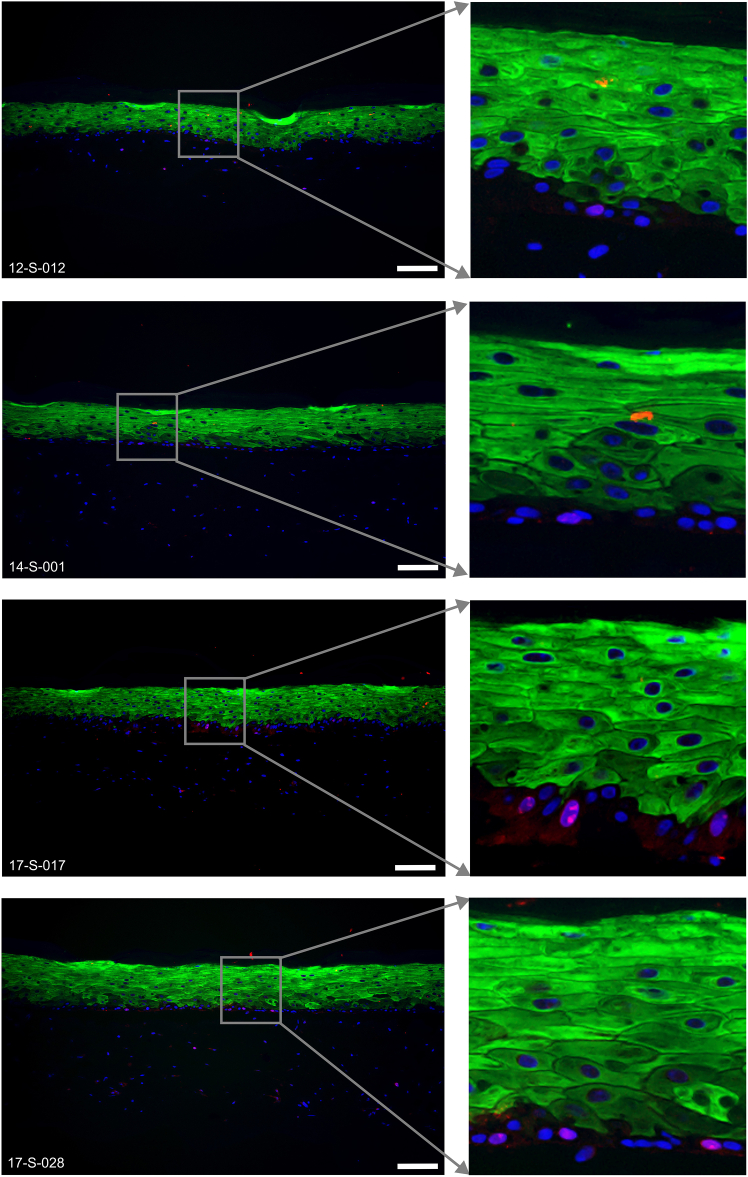
Representative immunofluorescence images of K10 (green) and Ki67 (red) expressed in the 6-well tissues 12-S-012, 14-S-001, 17-S-017 and 17-S-028 are the deidentified donor IDs. Scale bar, 100 μm.

**Figure 8:**
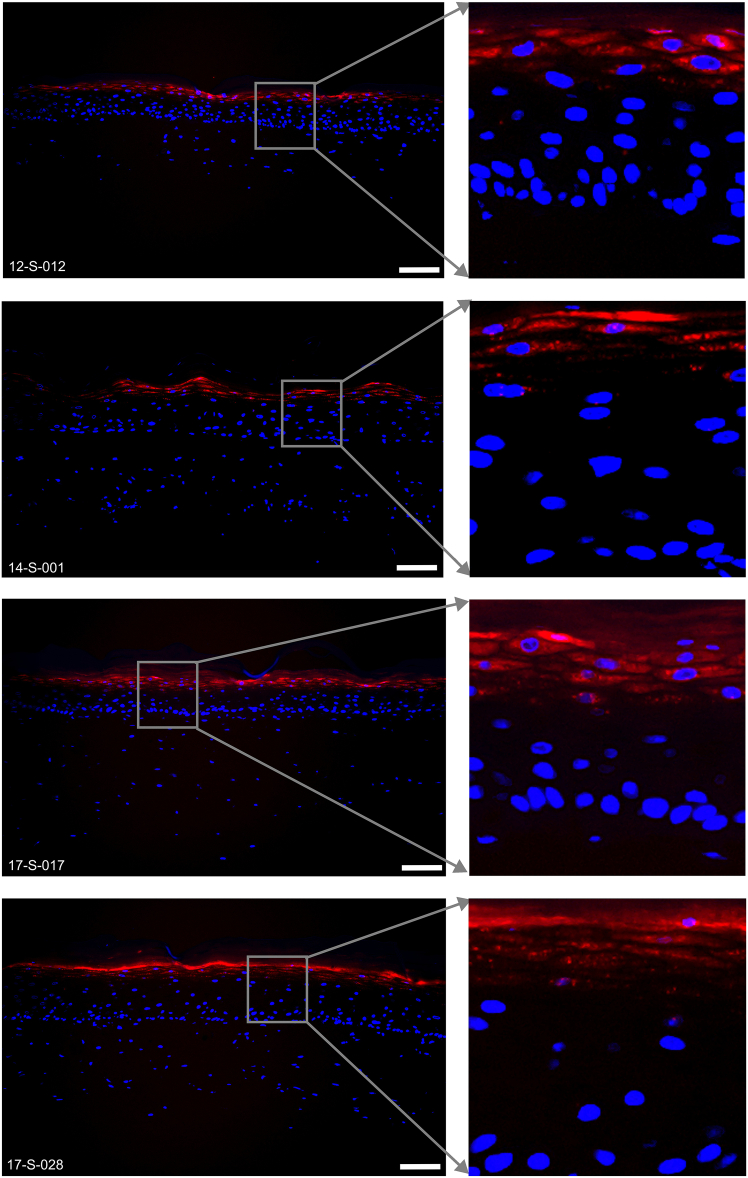
Representative immunofluorescence images of Filaggrin (red) expressed in the 6-well tissues 12-S-012, 14-S-001, 17-S-017 and 17-S-028 are the deidentified donor IDs. Scale bar, 100 μm.

**Figure 9:**
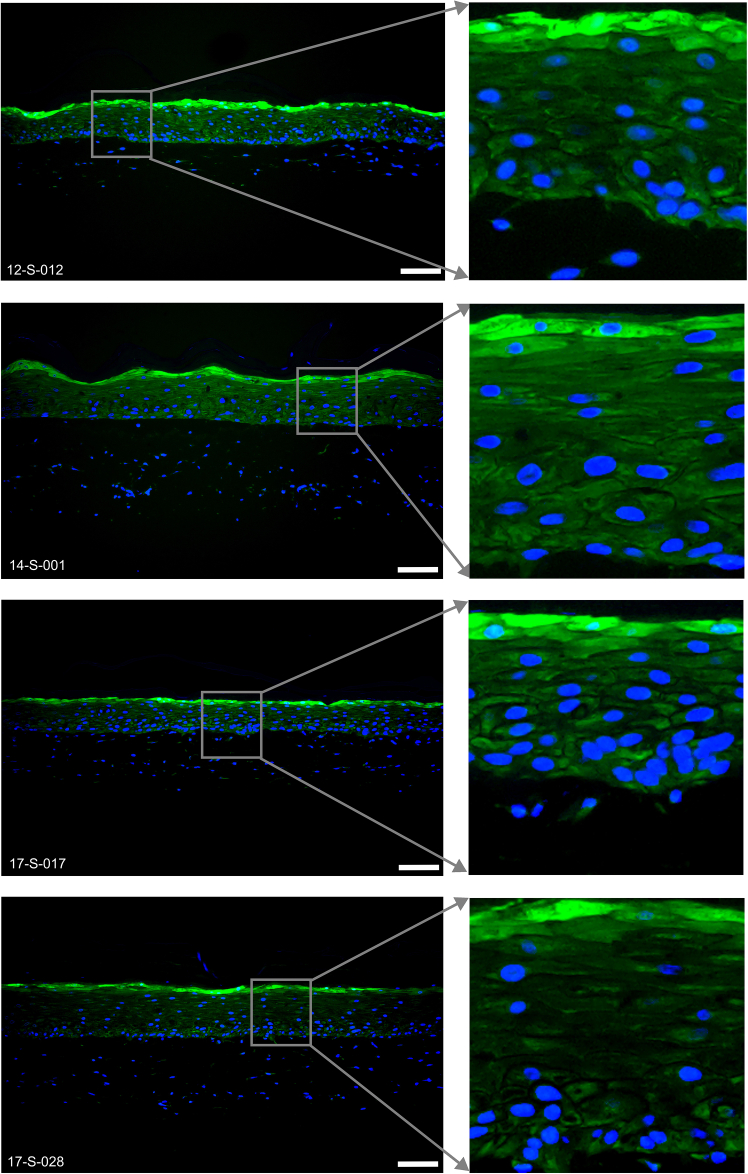
Representative immunofluorescence images of Loricrin (green) expressed in the 6-well tissues 12-S-012, 14-S-001, 17-S-017 and 17-S-028 are the deidentified donor IDs. Scale bar, 100 μm.

**Figure 10:**
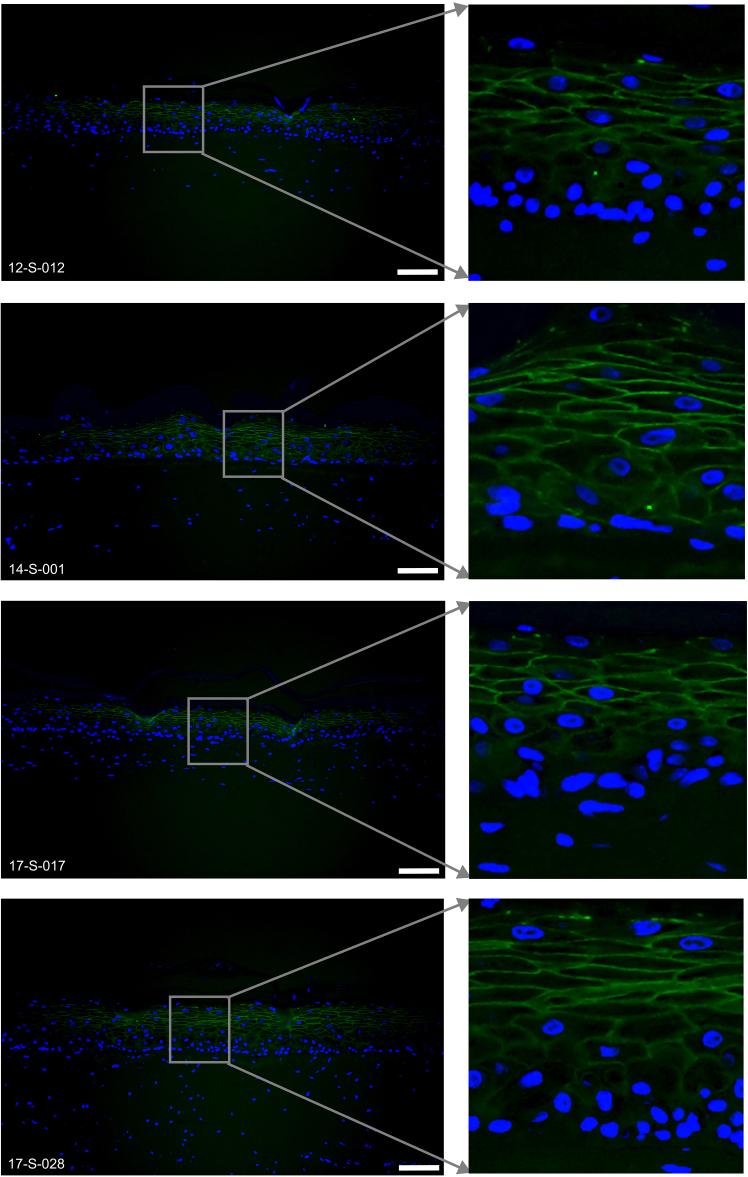
Representative immunofluorescence images of Claudin-1 (green) expressed in the 6-well tissues 12-S-012, 14-S-001, 17-S-017 and 17-S-028 are the deidentified donor IDs. Scale bar, 100 μm.

Keratinocytes and fibroblasts are separated in epidermal and dermal equivalent layers mimicking *in vivo* condition as indicated by the keratinocyte marker K14 and the fibroblast marker Vimentin (Figure 6). The suprabasal differentiation marker K10 marks the epidermal equivalent layer except the stratum basale (Figure 7). Skin barrier proteins like Filaggrin (Figure 8) and Loricrin (Figure 9) can be detected in the stratum granulosum layer. Cell-cell adhesion is intact as demonstrated by the tight junction marker Claudin-1 (Figure 10) expressed in suprabasal layers of the epidermis.

## Limitations

In this protocol, two major cell types were derived from the dermis and the epidermis of human skin, namely fibroblasts and keratinocytes. In the absence of additional cell types the entire skin is not represented. For example, the presented 3D skin model is not immune competent and is not pigmented. This model is suitable for studying innate immunity of keratinocytes, such as inflammasome, as demonstrated in our previous publications.^1^^,^^2^ In addition, studies related to paracrine signaling between fibroblasts-keratinocytes, crosstalk between microbiota-epidermis, skin barrier integrity, and extracellular matrix remodeling in the context of 3D microenvironment, may exploit the presented model to address biological hypothesis. To study inflammatory skin diseases and pigmentary disorders, the addition of relevant immune cells or exogenous cytokines, and the inclusion of melanocytes, respectively, are required.

The presented 3D skin model is not ideal for long-term culture, as we observed a great reduction of Ki67 expression in keratinocytes from the 5^th^ to the 14^th^ day of air lifting. This is due partly to the stochastic behavior of the maturing skin culture and also could be a reduced proliferative potential of the culture. Hence, it is likely that the epidermis of 3D model will contain predominantly corneum layers with additional weeks of air lifting. To enable long-term culture for studies related to skin aging, further identification and inclusion of exogenous ligands that promote keratinocyte stemness and proliferation may be necessary.

## Troubleshooting

### Problem 1

Excessive tissue contraction of 3D skin in 6-well format (Steps 30 and 33 at the “step-by-step method details” section).

### Potential solution

• After adding the acellular collagen I mixture and the collagen I-fibroblast mixture onto the 6-well inserts, tilt and rotate the 6-well plate while the mixtures are still in liquid form. This precaution measure increases the contact area between the mixtures and the insert wall. Hence, it can potentially reduce tissue contraction with more dermal attachment to the insert contour wall.
• Test matched fibroblasts and keratinocytes from alternative human donors. As demonstrated in Figure 2, skin cells derived from four donors show varying degree of contraction.

### Problem 2

Excessive tissue contraction of 3D skin in 12-well format (Step 29 at the “step-by-step method details” section).

### Potential solution

• Since tilting and rotation are not an option for the 3D skin in 12-well format due to small surface area, the inclusion of chitosan in the acellular collagen I matrix layer increases the mechanical strength of dermis. As a result, the acellular layer becomes stiffer and prevents the top cellular layer of dermis from excessive contraction.
• Test matched fibroblasts and keratinocytes from alternative donors. As demonstrated in Figure 2 for the 6-well model, skin cells derived from four human donors show varying degree of contraction and would also assist in the 12-well model.

### Problem 3

Air bubbles are generated during mixing steps of the dermal mixture. These bubbles are transferred into the dermis equivalent layer. Bubbles in the dermal equivalent layer disrupt final skin quality (Steps 29 and 32 at the “step-by-step method details” section).

### Potential solution

• After mixing all reagents, incubate the mixtures in ice for 5 min. Due to the viscous nature of collagen mixture, micro-bubbles float to the surface slowly.
• Remove bubbles attached to the external side of pipette tips.
• Use the recommended low liquid retention tips for handling viscous mixtures.
• Use reverse pipetting technique and maintain a slow flow speed to avoid generating additional bubbles.

### Problem 4

Large pipetting error during the preparation of mixtures with collagen I (Steps 29 and 32 at the “step-by-step method details” section).

### Potential solution

• Use the recommended low liquid retention tips for handling viscous mixtures.
• Prepare extra volume of the master mixtures with collagen I.
• Use reverse pipetting technique and maintain a slow flow speed.

### Problem 5

Mixture with collagen I becomes semi-solid before pipetting onto the inserts (Steps 29 and 32 at the “step-by-step method details” section).

### Potential solution

• Put a few pieces of dry ice at the bottom of the ice box to slow down ice melting and temperature increase.
• Keep mixtures with collagen I in ice throughout the preparation process.

## Resource availability

### Lead contact

Further information and requests for resources and reagents should be directed to and will be fulfilled by the lead contact, John E. A. Common (john.common@newcastle.ac.uk).

### Technical contact

Technical questions on executing this protocol should be directed to and will be answered by the technical contact, Khek-Chian Tham (khekchian_tham@asrl.a-star.edu.sg).

### Materials availability

For primary human fibroblasts and keratinocytes, please contact Carine Bonnard (carine_bonnard@asrl.a-star.edu.sg).

### Data and code availability

No new data and code were developed in this paper.

## Acknowledgments

This research was supported by funding from Agency for Science, Technology, and Research and A∗STAR-EDB-NRF IAF-PP grants (H17/01/a0/004, “Skin Research Institute of Singapore”; H18/01/a0/016, “Asian Skin Microbiome Program”; and H22/J1/a0/040, “Asian Skin Microbiome Program 2.0”) and Central Research Funding-Applied Translational Research (CRF-ATR). J.E.A.C. receives support from the NIHR Newcastle Biomedical Research Centre. We acknowledge Biorender for making some of the schematics.

## Author contributions

K.-C.T. wrote the manuscript. K.-C.T. and S.S.L. conducted the experiments. C.B. provided primary human fibroblasts and keratinocytes and reviewed the manuscript. J.E.A.C. and K.-C.T. conceptualized the project. J.E.A.C. reviewed, edited the manuscript, and supervised the work. All authors read and reviewed the manuscript.

## Declaration of interests

The authors declare no competing interests.
